# Habitat prioritization for bat conservation: A case study in Vietnam

**DOI:** 10.1371/journal.pone.0331094

**Published:** 2025-09-11

**Authors:** Le Quang Tuan, Vuong Tan Tu, Tran Anh Tuan, Vu Dinh Thong, Nguyen Truong Son, Nguyen Thanh Luong, Nguyen Tran Vy, Hoang Trung Thanh, Gábor Csorba, Tamás Görföl, Mao-Ning Tuanmu

**Affiliations:** 1 Department of Life Science, National Taiwan Normal University, Taipei, Taiwan; 2 Biodiversity Program, Taiwan International Graduate Program, Academia Sinica, Taipei, Taiwan; 3 Biodiversity Research Center, Academia Sinica, Taipei, Taiwan; 4 Institute of Biology, Vietnam Academy of Science and Technology (VAST), Hanoi, Vietnam; 5 Graduate University of Science and Technology, VAST, Hanoi, Vietnam; 6 Institute of Tropical Biology, VAST, Ho Chi Minh City, Vietnam; 7 University of Science, Vietnam National University, Hanoi, Vietnam; 8 Department of Zoology, Hungarian Natural History Museum, Budapest, Hungary; 9 National Laboratory of Virology, Szentágothai Research Centre, University of Pécs, Pécs, Hungary; Institut Pasteur de Madagascar, MADAGASCAR

## Abstract

Bats play a vital role in ecosystems through providing essential functions and services, such as pollination and pest control, but they face many threats from anthropogenic activities, climate change, and diseases. While establishing protected areas can be a conservation measure to mitigate bats’ population declines and habitats loss, the effectiveness of current protected areas in conserving bat species remains poorly understood. Using Vietnam as a case, this study aims to evaluate the representation of the existing protected area network for bats in terms of the proportion of their distribution ranges covered and the number of well-covered species. We also identified areas that would improve the representation using the spatial prioritization approach. Our results showed that while Vietnam’s protected area network provides better representation than random allocation, it currently only covers 6.12% of bat distribution ranges on average, leaving the distribution areas of more than 78 (96%) of the 81 species examined in this study insufficiently represented. Through the spatial prioritization, the forests in Central Highlands, western Central Coast, and Northwest were identified as the key regions for bats. Expanding the current network to cover 9% and 30% of the land under the national and global targets could improve the range coverage to 11.89% and 41.99%, respectively, reducing the number of poorly represented bats to 10–23 (12.3–28.4%) species. These findings offer critical insights for conservation practitioners and policymakers into prioritizing resources and efforts for improving conservation of not only bats in Vietnam but also biodiversity in general in other places.

## Introduction

Bats (Order Chiroptera) play important roles in various ecosystems and provide key services to humans, including pest suppression, seed dispersal, and pollination [1]. Additionally, bats serve as valuable indicators for biodiversity assessments due to their quick reactions to environmental changes [2]. However, bats face numerous pressures that threaten their survival. Specifically, the destruction and alteration of roosts and foraging habitats due to urbanization, agriculture, and deforestation are primary threats [3]. Other human activities, such as cave tourism, wind energy development, and bushmeat hunting, also disturb bat colonies, reducing their survival rates, and contributing to population declines [4]. Moreover, changes in climate patterns, such as rising temperatures, further threaten bat survival, particularly by disrupting their hibernation patterns, reducing prey availability, and altering habitats [5].

Establishing protected areas (PAs) is a primary tool for biodiversity conservation, but its lack of effectiveness is not uncommon around the world, especially in developing countries [6,7]. This phenomenon is often a consequence of inadequate management and lack of financial support due to poor governance quality and bureaucratic inefficiency [8]. However, it can also stem from the flaws in the design of PAs [9], especially the poor representativeness of PAs for targeted species or ecosystems [10]. For instance, a study on African bats found that only 5% of their suitable habitats are covered by protected areas [11]. Similarly, 67.6% of Brazil’s bat species have their range poorly protected by PAs [12]. This lack of representativeness is also common in other taxa [13]. Globally, only 4–9% of terrestrial amphibians, birds and mammals are sufficiently represented within the existing protected areas [14], and many PAs are biased toward protecting charismatic and well-known species, leaving less attention for inconspicuous taxa (e.g., small bodied vertebrates and invertebrates) [15]. The inadequate representativeness is particularly profound in tropical and lower income regions, although many of them are biodiversity hotspots [7].

The lack of representativeness in protected areas has been found to undermine biodiversity conservation efforts on reducing species extinction risk [16], preventing ecosystem services loss [17], and enhancing climate resilience [18], and thus result in the failure of global conservation strategies [7]. For example, bird populations in areas with limited PA coverage experience greater exposure to habitat destruction, hunting and other anthropogenic threats, compared to those with greater PA coverage, leading to increased extinction risk [16]. Additionally, underrepresented species and ecosystems may be more vulnerable to climate change if PAs do not include diverse and resilient habitats that allow species to move or adapt to changing conditions [18]. Accordingly, as a global target has been set to increase the protected lands to 30% by 2030 [19], it is crucial to ensure the increased coverage of PAs can effectively improve their biodiversity representativeness in order to safeguard biodiversity and maintain the health and functionality of ecosystems worldwide.

An integration of species distribution models (SDMs) and spatial conservation prioritization (SCP) is a powerful tool that can significantly improve the representativeness of protected areas [11]. SDMs can offer critical insights into where species currently exist, where species could potentially occur, and how species distributions might shift in response to environmental changes [20]. Based on this information, SCP uses data-driven approaches to identify areas that are most important for biodiversity conservation, ensuring that conservation efforts are placed in the areas which can maximize ecological benefits [21]. SCP has been applied in many cases to support and enhance conservation design [22]. For example, Sinclair *et al.* (2018) found that 74% of global conservation prioritizations have led to on-the-ground actions, demonstrating collaboration between the academic sector and practitioners [23]. Additionally, Lagabrielle *et al.* (2018) used a SCP method to identify priority conservation areas in Brazil’s Atlantic forests, significantly improving land allocation efficiency and reducing conflicts between development and conservation [24]. However, despite the availability of the powerful tools, they have seldom used for guiding bat conservation, especially in the tropics, because spatial information on bat distributions is often unavailable, incomplete or inaccurate [3,25].

Vietnam, home to approximately 30% of Asia’s bat fauna and 10% of the global bat fauna, is a crucial region for bat diversity [26]. It harbours three endangered and five vulnerable species, as listed in the IUCN Red List of Threatened Species [27]. Bats in Vietnam are regarded as important indicators of overall biodiversity, both for their significant representation within the mammal fauna and their role as keystone species providing essential ecosystem services [28,29]. However, several bats in Vietnam are at risk of extinction due to habitat loss, disturbance, and overhunting [29,30]. While PAs protect critical habitats for many wildlife species in Vietnam [28,31], the extent to which these areas represent suitable habitats for bats remain poorly understood. To fill this gap, this study aims to (i) assess the representativeness of the current protected area network in Vietnam for bats and (ii) identify priority areas for expanding the network to align with national and global PA coverage target.

## Materials and methods

### Study area

**Fig 1:**
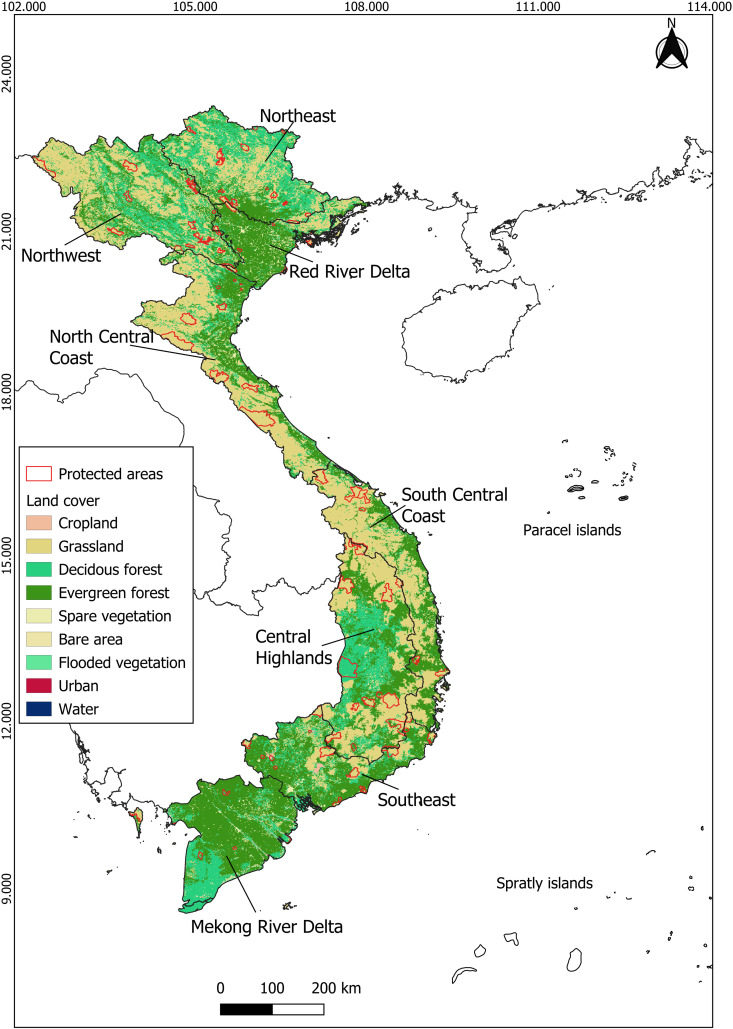
Land cover and the locations of current protected areas in Vietnam. The information on the land cover and the protected areas were obtained from the ESA CCI land cover 2018 product [33], and the World Database on Protected Areas [34]. **Copyright notice**: © ESA Climate Change Initiative – Land Cover project 2017; © UNEP-WCWC and IUCN – WDPA 2023.

Vietnam, located in Southeast Asia, spans a considerable latitudinal range (Fig 1). It exhibits a significant temperature gradient, with the annual mean temperature increasing from 19^o^C in the northern regions to 27^o^C in the south. Annual precipitation also varies widely, ranging from under 800 mm in the coastal southeast to nearly 5,000 mm in the mountainous northeast, characterized by distinct wet and dry seasons. Generally, the degree of seasonality in both temperature and precipitation diminishes from north to south [32].

### Bat occurrence data

In total, 8431 unique occurrence records of 116 bat species in Vietnam were compiled from various sources (S1 Table). Of these, 65.4% were obtained from the Global Biodiversity Information Facility (GBIF; downloaded on June 3rd, 2023, DOI: 10.15468/dl.28jn5y), 26.6% from published literature, 5.1% from unpublished records of the Institute of Ecology and Biological Resources (IEBR), 1.0% from unpublished records in the Hungarian Natural History Museum, and 1.7% from our field notes. The number of records per species ranges from 21 for *Murina annamitica* to 838 for *Pteropus vampyrus*. Detailed information on the data sources and the data searching and cleaning approaches is provided in Tuan *et al.* (2023) [35].

### Environmental parameters

#### Climate data.

We obtained 19 bioclimatic variables from CHELSA (Version 2.1). These variables were derived from the mean monthly maximum and minimum temperatures and mean monthly precipitations between 1979 and 2013 [36,37]. Compared to other products (e.g., WorldClim), CHELSA provides more accurate predictions of precipitation patterns and yields better species range predictions [36]. To address the multicollinearity among the variables, we selected one from highly correlated variables (Spearman ρ≥0.7) based on their biological or ecological relevance to bat distributions. Six variables which have been found to influence the reproductive success, mortality rates, activity patterns, roost selection, and/or torpor-arousal cycles of bats [30,38,39], including the mean temperatures in the warmest and coldest quarters of the year, mean diurnal temperature range, annual precipitation, and precipitations in the warmest and coldest quarters, were selected for building species distribution models (S1 Fig).

#### Land cover data.

We obtained the ESA CCI Land Cover Product [33] for the year of 2018 from ArcGIS REST Services (https://env1.arcgis.com/arcgis/rest/services/esa_cci_landcover_2018/ImageServer, accessed on Apr 2024). This product classified the global land cover into 10 types at a 300 m resolution with the World Cylindrical Equal Area projection. We calculated the percent cover of each land cover type within every 1-by-1 km grid cell with the same projection across the study area. We only used the percent cover of cropland, deciduous forest, evergreen forest, flooded vegetation, and grassland/scrub to build species distribution models (see below) because other land cover types cover a small proportion of the study area.

#### Karst data.

Given that karst areas provide caves as roosts for many bat species in Vietnam [29], we also included the distribution of karst as a variable in our species distribution models. We obtained a global data layer with polygons delineating areas with carbonate rocks from the World Map of Carbonate Rock Outcrops V3.0 (https://digital.lib.usf.edu/SFS0055342/00001). Since most carbonate rocks are prone to karstification, these areas can be considered as potential karst regions [40]. However, it is important to note that not all areas with carbonate rocks provide suitable caves for bats, introducing some uncertainty in this data layer. We converted this data into presence or absence of karst at 1-by-1-km resolution by rasterizing it with the World Cylindrical Equal Area projection.

#### Species distribution modelling.

We built species distribution models with the above-mentioned bat occurrence records and the climate, land-cover and karst data for the 81 bat species that had more than 20 occurrence records (S1 Table). All environmental data were re-projected to the World Cylindrical Equal Area projection at the 1-by-1 km resolution. We built models for individual species using the Maxent (ver. 3.4.1; [41,42]) with the target-background approach [43], which used all of the presence locations of all the 116 species as the background. We used Maxent because it does not require absence data and consistently outperforms many other modelling algorithms [44,45]. We assessed model performance using a 5-fold spatial block cross-validation [46] with the values of the area under the receiver operating characteristic curve (AUC) and the True Skill Statistic (TSS). The model configuration that achieved the highest AUC value across all 81 species—characterized by a regularization multiplier of 2.5 and a combination of linear, quadratic, and hinge feature types (S2 Fig) —was used to build the final models. To maximize the information used for predicting species distribution, we included all presence records of a species to build the final model. The continuous model outputs (i.e., predictions of species presence probabilities) were converted into a binary format (i.e., presence/absence of the species) with the threshold determined by the 10^th^ percentile of the training presences [47] because this approach does not require absence data. The model generation and validation were conducted in R (version 4.2.2) with the *dismo* (version 1.1.4; [48] and *blockCV* ([49]) packages. Detailed modelling approaches are available in Tuan *et al.* (2023) [35].

#### Spatial prioritization analyses.

We quantified the importance of every1-by-1-km cell in Vietnam for bat conservation and identified the priority areas with different PA coverage targets using Zonation [50]. Zonation ranks all cells in a study region by their conservation values, quantified by the complementarity and irreplaceability of the species occurring in each cell, and then removes the cells with the lowest ranks [50]. It repeats this process iteratively until the remaining cells achieve the conservation target set by the user. In this study we employed the Additive Benefit Function (ABF) cell-removal rule, which balances multiple species in systematic conservation planning (Moilanen, 2007). The conservation value for the cell *i* (*δi*) was calculated as:


$$\delta_{i}=\frac{1}{c_{i}}w_{j}\sum_{j}\Delta V_{j}+\;\beta *\Delta \left(\frac{BL}{A}\right)=\;\frac{1}{c_{i}}w_{j}\sum_{j}\left[V_{j}\left(q_{j}\right)-V_{j}\left(q_{j}-i\right)\right]+\;\beta *\Delta \left(\frac{BL}{A}\right),$$

where *qj* indicates the representation of species j in remaining cells, *qj-i* indicates the representation of species *j* of the remaining cells excluding cell *i*, and thus Δ*Vj* indicates the loss of the representation of species *j* when cell *i* is removed; *wj* is the weight of the species *j* with a higher value indicating higher conservation importance; *ci* is the cost for including cell *i* into the remaining cell patches; $\Delta \left(\frac{BL}{A}\right)$ is the change in the ratio of the boundary length to the area of the remaining cell patches following the removal of cell *i*; and β is a constant that defines the strength of the boundary length penalty (BLP) with a higher value leading to a lower edge-to-area ratio of remaining areas thus increasing the compactness and ecological connectivity among prioritized conservation areas. Therefore, a higher *δi* value indicates that cell *i* is more worthy of protection relative to the associated cost.

We weighted the species based on their IUCN Red List category with a value of 1 for least-concern species, 2 for near threatened species, 3 for vulnerable species, 4 for endangered species, and 5 for critically endangered species, following Kullberg *et al.* [51]. We assigned a cost value, as proxy for land acquisition cost, for a cell based on its land cover with a value of 10 for nature land cover types (e.g., evergreen forest) and a value of 1 for human-associated land cover types (e.g., urban), following Whitehead *et al.* [52]. In each iteration, we used the “edge removal” function to remove lowest-ranked cells from the edges of remaining cell patches to maintain the connectivity of remaining cells as high as possible [53]. In the prioritization process, we also used the approach of boundary length penalties (BLP), which imposed a higher penalty to a higher perimeter-to-area ratio of the remaining cell patches, to increase the cohesion of the patches [54]. We set the BLP as 0.1, which means that the conservation value of cell i was add a value equal to the change of ratio of boundary length to area of the reserve network following removal of cell i, multiple by 0.1. To reduce the computation time, we removed 50 lowest-ranked cells in each iteration. We assigned the highest conservation value to the cells within current PAs to ensure their inclusion in the prioritized areas. The boundaries of the current PAs were obtained from the World Database on Protected Areas [34]. We included the PAs of the IUCN categories from I (strict nature reserve) to VI (protected areas with sustainable use of natural resources) (S3 Table).

We considered two conservation targets in the prioritization process. First, according to the Vietnam National Strategy on Biodiversity to 2030, vision 2050 [55], protected areas are expected to cover 9% of the land area by 2030. Second, aligning with the Target 3 of the Kunming-Montreal Global Biodiversity Framework of the Convention on Biological Diversity, which aims to increase the coverage of protected areas and other effective area-based measures to 30% by 2030 [56], we also set a target of 30% land coverage. Since the current protected areas (covering ~5% of land in Vietnam) were always included in the prioritized areas, we identified additional 4% and 25% of areas to achieve the national and global conservation targets, respectively.

#### Area representativeness for bats.

We quantified the representativeness of current protected areas and the prioritized areas from Zonation for bats in Vietnam using two approaches. First, we calculated the proportion of the distribution range of each bat species covered by the protected areas or prioritized areas [57], and then calculated the average across all 81 species. Second, we measured the representativeness using the number of bat species whose distribution ranges were well-represented by the current protected areas or the prioritized areas. The term “well-represented” was defined by following the approach outlined by González-Maya *et al.* (2015) [58] but adapting it to bats in Vietnam. Specifically, species with restricted distribution (range size <1,000 km^2^), were considered as well-represented if 100% of their range was covered by protected areas or prioritized areas; those with wide distribution (range size > 25,000 km^2^), were considered as well-represented if at least 10% of their range was covered; and for those in between, the coverage criterium was determined by a linear interpolation between 10% and 100% based on the range size.

## Results

### Predictions of bat distributions

The mean AUC value of the distribution models was 0.770 ± 0.102 (mean ± SD), with a value greater than 0.7 for 61 (70.3%) of the 81 bat species modelled (S2 Fig, S1 Table). The mean TSS value of the distribution models was 0.495 ± 0.184 (mean ± SD), with 54 models (66.7%) showing values greater than 0.4 (S1 Table). These results indicated that most models performed well. We present the results with all 81 species included in the main text and the results including only the 61 species with an AUC value ≥0.7 in the Supplementary Materials, because the results showed similar patterns (see below). Although the contributions of predictor variables in the model varied among species, the mean temperature in the coldest quarter was the most important factor determining bat species distributions, followed by the evergreen forest coverage and precipitation in the coldest quarter (S3 Fig, S2 Table).

**Fig 2:**
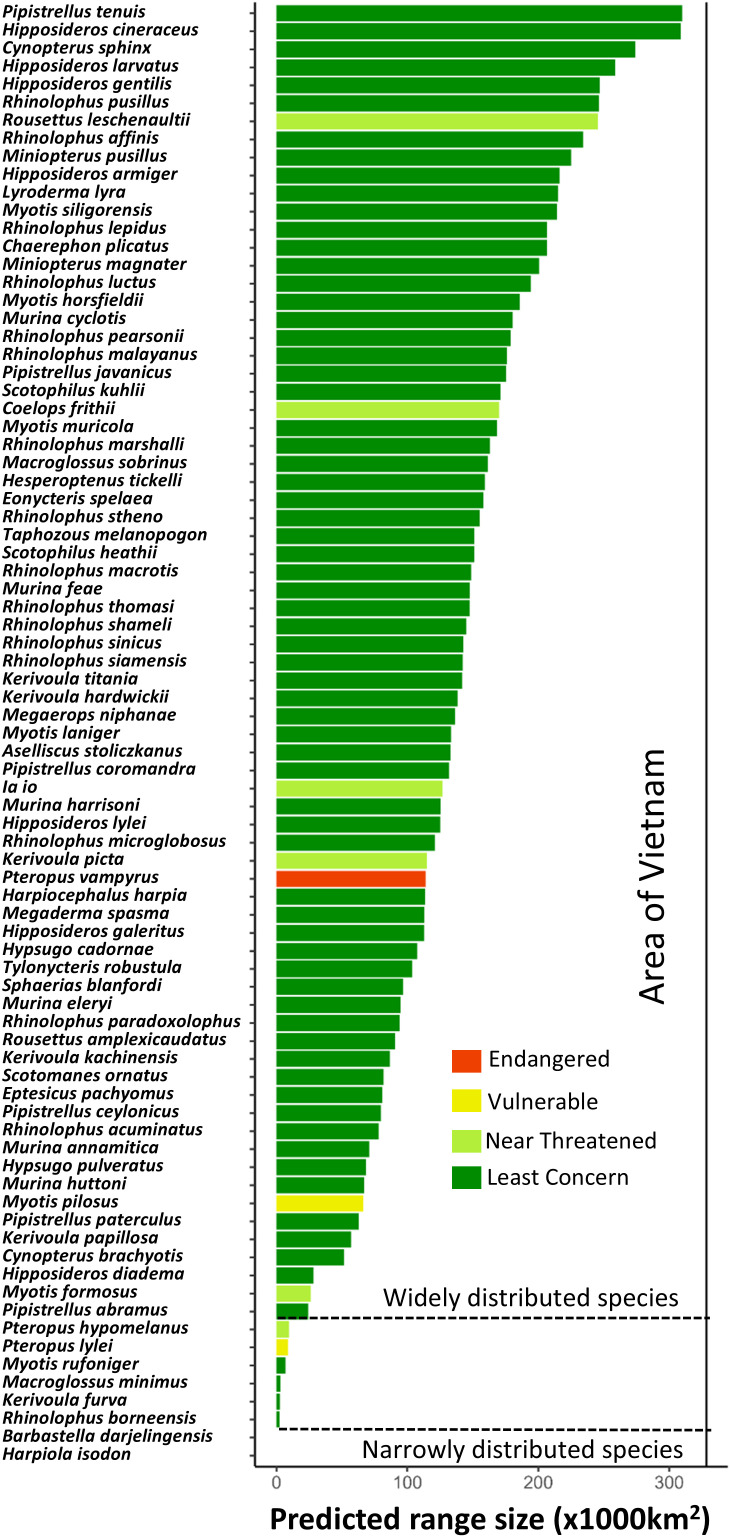
Predicted distribution range size for the 81 bat species modelled. Species ordered by their range size within Vietnam.

The predicted distribution ranges of bat species within Vietnam varied significantly in size, ranging from 33 to 309,825 km^2^ (Fig 2). Most taxa have a wide distribution (i.e., range size > 25,000 km^2^), and only two species, *Harpiola isodon* and *Barbastella darjelingensis* had a restricted distribution (i.e., range size < 1,000 km^2^) (Fig 2).

### The representativeness of current protected areas for bats

**Fig 3:**
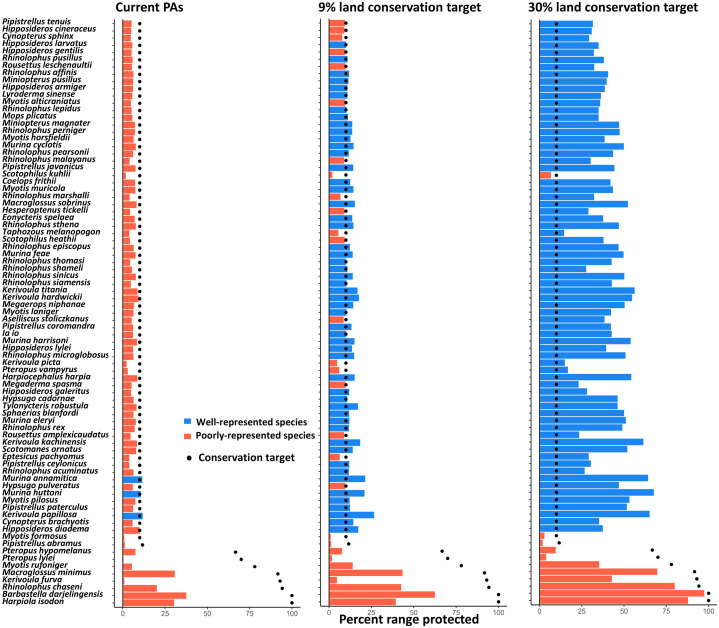
Representativeness of current protected areas, and the prioritized areas with the conservation targets of 9% and 30% land coverage. The representativeness was measured as the proportion of a species’ distribution range covered by the protected areas or prioritized areas. The dots indicate the criterium for a species being considered as a well-represented species. The species are ordered by their current range size within Vietnam.

The current protected areas (PAs) covered, on average, 6.12% (range: 0.42%−37.50%) of the distribution range of the bat species within Vietnam (Fig 3). The mean coverage was higher than the land coverage of current PAs in Vietnam (i.e., 5%), indicating that the current PAs had greater representativeness for bats compared to randomly placed PAs. However, 78 of the 81 bat species (~96.3%), including all threatened species (i.e., endangered and vulnerable species), had their range poorly represented by the current PAs (Fig 3). The representativeness for threatened species was particular low with only 3.05%, 7.34%, and 0.42% of the range being protected for *Pteropus vampyrus*, *Myotis pilosus* and *Pteropus lylei*, respectively (Fig 3). The results for the 61 species with models’ AUC higher than 0.7 are similar with those for all 81 species, with 6.64% (range: 0.01%−13.95%) of the distribution range covering by the PAs and 48 of the 61 bat species (~96.3%) having their range poorly represented (S5 Fig).

### Representativeness of prioritized areas for bats

**Fig 4:**
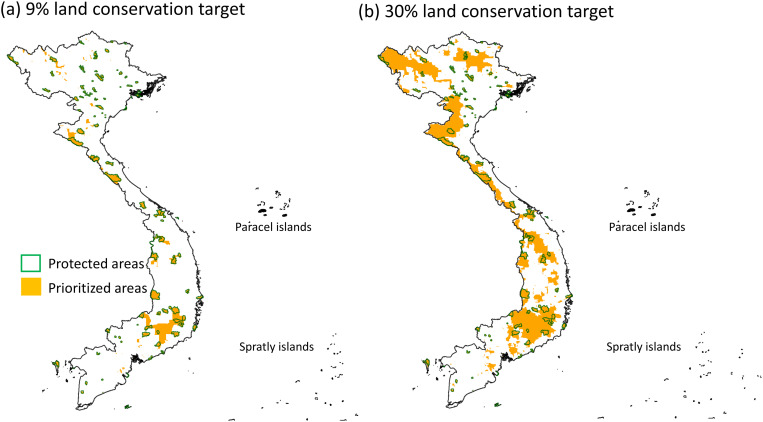
Distribution of the prioritized conservation areas for bat species in Vietnam identified by the spatial prioritization analysis. Nine percent (a) and 30% (b) of the land area with the highest priority were identified corresponding to the national and global targets, respectively. The boundaries of current protected areas in Vietnam are also shown. The protected area boundaries were obtained from the World Database on Protected Areas [34]. Copyright notice: © UNEP-WCWC and IUCN – WDPA 2023.

With the target of 9% land coverage (i.e., national target), the spatial prioritization analysis indicated that some evergreen forest areas in Central Highlands and western Central Coast should be added to the PA network to improve the representativeness for bats (Fig 4a). The priority areas covered 11.89% (range: 0.95%−62.50%) of the distribution range of the bat species (Fig 3). Additionally, 58 species (71.6%), including one of the three threatened species, had their range well-represented by the priority areas (Fig 3). These results indicated that a 79.28% increase (from 5% to 9%) in the size of the PA network can lead to a 94.28% (from 6.12% to 11.89%) to 17.6-fold (from 3 to 58 species) increase in the representativeness for bats.

With the target of 30% land coverage (i.e., global target), the spatial prioritization analysis indicated that in addition to the Central Highlands and western Central Coast, the forests in Northwest are also important for bat representation (Fig 4b). The identified priority areas covered 41.99% (range: 1.86%−97.50%) of the distribution range of the bat species (Fig 3). Additionally, 71 species (87.6%), including one of the three threatened species, had their range well-represented by the prioritized areas (Fig 3). These results indicated that a 6-fold increase (from 5% to 30%) in the size of the PA network can lead to a 7-fold (from 6.12% to 41.99%) to 22.6-fold (from 3 to 71 species) increase in the representativeness for bats. The prioritization results for only the 61 species with models’ AUC higher than 0.7 are similar (S5 and S6 Figs).

## Discussion

Bats provide important ecosystem services and are threatened by various human-induced environmental changes [5,11], but they are rarely considered as a conservation target in the process of establishing protected areas (PAs) [59]. The effectiveness of current PAs for bats has been poorly studied, especially in tropical countries like Vietnam [12]. This study presents the first assessment of the representativeness of current PAs for protecting bats in Vietnam and identifies the priority areas for improving the representativeness under both national and global conservation targets. Our results show that while the current PAs are more representative than randomly placed PAs, they are far from sufficient to protect bat habitats, suggesting a need of establishing more protected areas. By integrating the spatial information of bats’ distributions and the spatial conservation prioritization approach, we also show that the country’s current plan to expand the protected area network to 9% of land coverage by 2030s has the potential to significantly increase the representativeness. However, to ensure that the network can well cover distribution ranges of a majority of bat species in Vietnam, achieving the global target (i.e., protecting 30% of the land by 2030s) should be the goal. Our identification of the priority areas provides essential information for conservation planning. The findings also highlight the need to adjust current conservation strategies to ensure the effective protection of bat populations and their habitats.

In this study, we found that the protected areas in Vietnam exhibit low representativeness for bat distribution ranges. The representativeness was similar to that for bats in Brazil (6.8%) [12] and lower than that for many other taxa, including global terrestrial mammals (20%) [57] and mammals in Costa Rica (28.3%) [58]. The low representativeness of protected areas could be attributed to the good flying ability of bats, similar to birds [60], leading to a large distribution range [12]. The low representativeness may also result from the fact that bats are usually not the conservation target during the protected area design in Vietnam [61], like in other countries [62].

Despite being a hotspot for bat diversity, our findings reveal that Vietnam’s current protected areas network is insufficient in safeguarding bats. Many previous studies have highlighted the necessity of expanding protected areas to prevent long-term major losses of species diversity in Vietnam [61,63]. In response, Vietnam is working towards the National Biodiversity Strategy to 2030 and the vision for 2050, aiming to protect 9% of the country’s territory [55]. Considering both the national and global targets on land protection, spatial prioritization provides crucial information on where to expand current protected areas. Protecting these prioritized conservation areas can significantly enhance the representation of bats within the protected area network.

According to the spatial prioritization, the forest in Central Highlands, Northwest and western Central Coastal are the key regions to improve bat representation. Locations of the priority conservation areas are consistent with those designated for protecting Vietnamese mammals in general [64]. Most of these areas have been recognized as biodiversity hotspots in Vietnam. In particular, the forests in the Central Highlands are one of the most biodiversity rich area in Vietnam, hosting animals and plants from diverse biogeographical regions in Asia [65,66]. Northwest and Western Central Coast are situated in the transition zone of the Greater Annamites Ecoregion, one of the World Wildlife Fund’s Global 200 ecoregions and known for its exceptional biodiversity values [67]. However, rapid deforestation is threatening biodiversity in these regions [68], indicating a urgent need of protecting these areas.

Threatened and narrowly distributed bat species, such as *Coelops frithii, Ia io, Kerivoula picta, Myotis formosus, Myotis pilosus, Pteropus hypomelanus, P. lylei, P. vampyrus and Rousettus leschenaultii*, particularly need greater conservation efforts. These species face numerous threats, including habitat loss, climate change, and human disturbance [29,35]. Moreover, our findings suggest that current protected areas provide insufficient protection for these species according to their particular low representation in PAs (Figs 2 and 3). This suggests that their extinction risk might be higher than previously estimated, indicating the need for greater conservation investments, particularly outside current PAs, to effectively safeguard these bats.

Although this study considers all bats in Vietnam, the same approach can be applied to a subset of the species (e.g., cave-dwelling species or forest specialists) to guide specific measures for various conservation goals. Furthermore, it can be applied to other aspect of biodiversity, such as phylogenetic and functional diversity [9], as well as to other taxa and other conservation targets. By integrating multiple taxa and conservation targets into the prioritization process, we can create a more comprehensive conservation strategy that reflects the complex interdependencies within ecosystems. Expanding the approach to include a wider range of species and ecological functions would ensure that conservation efforts are more holistic, improving outcomes for both species and overall biodiversity [69].

The conservation value of different species is inherently context-dependent and can vary based on the specific conservation goals being pursued [70]. For instance, if the objective is to preserve evolutionary distinctiveness or functional traits that support ecosystem resilience, species prioritization may differ from approaches focusing solely on extinction risk [71]. In this study, species were weighted according to their IUCN Red List categories, which emphasizes the urgency of protecting threatened species. However, alternative weighting strategies could be employed in future applications to better reflect goals such as safeguarding ecosystem services, maintaining ecological interactions, or preserving phylogenetic diversity. Therefore, it is important for conservation planners to adapt the weighting scheme according to the intended conservation outcomes.

Our study relied on Maxent, a widely adopted algorithm known for its strong performance with presence-only data [41,42], for modelling species distributions. However, like all other algorithms, Maxent operates under specific assumptions and modelling decisions, such as background point selection, regularization settings, and feature types, which can introduce uncertainty into the resulting species distributions [41,43]. While we carefully evaluated various modelling settings and used the best one in this study, future studies could incorporate multiple SDM algorithms to better capture model uncertainties.

In conclusion, this study presents the first attempt to evaluate and improve the representativeness of protected areas for bat conservation in Vietnam. Our results show that current protected areas fall short in adequately safeguarding bats. However, the spatially explicit information on priority conservation areas obtained from an integration of species distribution modelling and spatial conservation prioritization offers a solution by optimizing the design of a protected area network. These findings provide great contributions to the conservation of not only bats but also broader biodiversity.

## Supporting information

S1 Table Bat species included in the study. The Red List category of the species, the number of occurrence records used in modeling, the model performance measures (i.e., AUC and TSS values), the predicted range size, and estimated range representations are also shown. The taxonomy and nomenclature used follow the Mammal Diversity Database (Mammal Diversity Database. (2024). Mammal Diversity Database (Version 2.0) [Data set]. Zenodo. https://doi.org/10.5281/zenodo.15007505), with updated names according to recent taxonomic revisions in parentheses.
(PDF)

S2 Table The contributions of environmental variables in the model of individual species. The value measures an environmental variable’s share in the model’s overall gain (a measure of fit). It’s calculated by summing each variable’s incremental gain during the model’s training process and converting these sums to percentages.
(PDF)

S3 Table The number and total area of protected areas in Vietnam by the IUCN category. The values were calculated from the data in the World Database on Protected Areas. The corresponding national categories are also provided.
(PDF)

S1 Fig Pairwise correlations of the six bioclimatic variables used for modeling the distributions of Vietnamese bat species. The values shown are Spearman’s rank correlation coefficients.
(PDF)

S2 Fig The AUC values of the distribution models built for individual bat species with different combinations of feature types and regularization values. Each boxplot shows the minimum, 25^th^ percentile, median, 75^th^ percentile and maximum values across all species modeled in the study. The boxplots and associated combinations are ordered by the AUC values, with those on the top having the highest value. The feature types are linear (L), quadratic (Q) and hinge (H).
(PDF)

S3 Fig AUC values of the distribution models built for the bat species in Vietnam. The red line shows the value of 0.7.
(PDF)

S4 Fig The percent contributions of the environmental variables across the bat species distribution models. Each boxplot shows the minimum, 25^th^ percentile, median, 75^th^ percentile and maximum values across all species modelled in the study. The variables are ordered by the medians of the percent contributions, with the one on the top having the highest median.
(PDF)

S5 Fig Representativeness of current protected areas, and the prioritized areas with the conservation targets of 9% and 30% land coverage. Same as Fig 3, but for bat species with an AUC value greater than 0.7.
(PDF)

S6 Fig Distribution of prioritized conservation areas for bat species in Vietnam identified by the spatial prioritization analysis. Same as Fig 4, but for the bat species with an AUC value greater than 0.7. The protected area boundaries were obtained from the World Database on Protected Areas [34]. Copyright notice: © UNEP-WCWC and IUCN – WDPA 2023.
(PDF)
