# Supplementary material for: Habitat prioritization for bat conservation: A case study in Vietnam
Source: PLoS One. 2025 Sep 11;20(9):e0331094. doi: 10.1371/journal.pone.0331094 (PMC12425236; doi:10.1371/journal.pone.0331094)
Supplement: S1 Table — The Red List category of the species, the number of occurrence records used in modeling, the model performance measures (i.e., AUC and TSS values), the predicted range size, and estimated range representations are also shown. The taxonomy and nomenclature used follow the Mammal Diversity Database (Mammal Diversity Database. (2024). Mammal Diversity Database (Version 2.0) [Data set]. Zenodo. https://doi.org/10.5281/zenodo.15007505), with updated names according to recent taxonomic revisions in parentheses. (PDF) [file pone.0331094.s001.pdf]

Table S1. Bat species included in the study. The Red List category of the species, the number of occurrence records used in modeling, the model performance measures (i.e., AUC and TSS values), the predicted range size, and estimated range representations are also shown. The taxonomy and nomenclature used follow the Mammal Diversity Database (Mammal Diversity Database. (2024). Mammal Diversity Database (Version 2.0) [Data set]. Zenodo. https://doi.org/10.5281/zenodo.15007505), with updated names according to recent taxonomic revisions in parentheses.

**Family**	**Species**	**IUCN Red list Category**	**Number of occurrence records**	**AUC**	**TSS**	**Range size within Vietnam (km^2)**	**Range size within current PAs (km^2)**	**Range size within 9% land conservation target (km^2)**	**Range size within 30% land conservation target (km^2)**
EMBALLONURIDAE	*Taphozous melanopogon*	Least Concern	74	0.783	0.600	151.161	5.655	8.346	22.103
HIPPOSIDERIDAE	*Aselliscus stoliczkanus*	Least Concern	99	0.850	0.578	132.921	7.078	11.550	51.126
HIPPOSIDERIDAE	*Coelops frithii*	Near Threatened	39	0.720	0.362	170.282	12.349	21.290	71.509
HIPPOSIDERIDAE	*Hipposideros armiger*	Least Concern	359	0.766	0.254	216.229	13.360	23.489	83.632
HIPPOSIDERIDAE	*Hipposideros cineraceus*	Least Concern	93	0.597	0.274	308.997	15.307	28.127	95.512
HIPPOSIDERIDAE	*Hipposideros diadema*	Least Concern	268	0.855	0.597	28.397	2.839	4.910	10.632
HIPPOSIDERIDAE	*Hipposideros galeritus*	Least Concern	78	0.775	0.527	112.854	5.666	13.525	31.732
HIPPOSIDERIDAE	*Hipposideros gentilis*	Least Concern	28	0.788	0.545	247.039	12.956	23.426	79.390
HIPPOSIDERIDAE	*Hipposideros larvatus*	Least Concern	244	0.633	0.264	258.681	14.418	26.097	90.436
HIPPOSIDERIDAE	*Hipposideros lylei*	Least Concern	55	0.693	0.418	125.102	7.613	17.010	49.374

MEGADERMATIDAE	*Lyroderma lyra (L. sinese)*	Least Concern	58	0.745	0.408	215.290	12.165	23.478	78.149
MEGADERMATIDAE	*Megaderma spasma*	Least Concern	189	0.753	0.458	113.028	5.903	10.970	26.113
MINIOPTERIDAE	*Miniopterus magnater*	Least Concern	35	0.660	0.318	200.625	14.589	27.548	94.497
MINIOPTERIDAE	*Miniopterus pusillus*	Least Concern	33	0.585	0.291	224.920	13.778	25.748	89.240
MOLOSSIDAE	*Chaerephon plicatus*	Least Concern	25	0.568	0.369	206.677	11.810	23.173	72.227
PTEROPODIDAE	*Cynopterus brachyotis*	Least Concern	385	0.825	0.628	51.692	2.986	7.429	18.229
PTEROPODIDAE	*Cynopterus sphinx*	Least Concern	289	0.676	0.228	274.029	12.945	21.416	80.490
PTEROPODIDAE	*Eonycteris spelaea*	Least Concern	163	0.727	0.321	158.288	11.123	21.721	59.553
PTEROPODIDAE	*Macroglossus minimus*	Least Concern	243	0.844	0.662	3.018	0.926	1.311	2.104
PTEROPODIDAE	*Macroglossus sobrinus*	Least Concern	49	0.637	0.475	161.358	13.036	24.625	84.421
PTEROPODIDAE	*Megaerops niphanae*	Least Concern	42	0.731	0.461	136.298	8.882	19.481	68.545
PTEROPODIDAE	*Pteropus hypomelanus*	Near Threatened	43	0.854	0.793	9.680	0.724	0.747	0.932
PTEROPODIDAE	*Pteropus lylei*	Vulnerable	74	0.966	0.881	8.795	0.037	0.155	0.344
PTEROPODIDAE	*Pteropus vampyrus*	Endangered	838	0.880	0.336	113.889	3.479	6.881	19.151
PTEROPODIDAE	*Rousettus amplexicaudatus*	Least Concern	198	0.793	0.541	90.532	4.170	8.074	21.219
PTEROPODIDAE	*Rousettus leschenaultii*	Near Threatened	56	0.491	0.219	245.435	13.241	23.902	79.452
PTEROPODIDAE	*Sphaerias blanfordi*	Least Concern	30	0.805	0.528	96.535	6.164	11.488	48.245
RHINOLOPHIDAE	*Rhinolophus acuminatus*	Least Concern	44	0.818	0.675	78.073	4.937	9.169	20.884

RHINOLOPHIDAE	*Rhinolophus affinis*	Least Concern	222	0.614	0.270	234.341	15.007	27.874	94.993
RHINOLOPHIDAE	*Rhinolophus borneensis*	Least Concern	39	0.892	0.816	2.325	0.468	0.990	1.859
RHINOLOPHIDAE	*Rhinolophus lepidus*	Least Concern	132	0.690	0.193	206.736	10.860	21.207	72.394
RHINOLOPHIDAE	*Rhinolophus luctus (R. periniger)*	Least Concern	120	0.628	0.380	194.436	14.043	26.544	92.170
RHINOLOPHIDAE	*Rhinolophus macrotis (R. episcopus)*	Least Concern	58	0.674	0.347	148.566	9.565	18.350	69.502
RHINOLOPHIDAE	*Rhinolophus malayanus*	Least Concern	121	0.778	0.444	175.754	6.942	15.547	53.181
RHINOLOPHIDAE	*Rhinolophus marshalli*	Least Concern	32	0.768	0.485	162.887	6.632	11.041	52.624
RHINOLOPHIDAE	*Rhinolophus microglobosus*	Least Concern	73	0.768	0.548	120.901	7.684	17.907	61.495
RHINOLOPHIDAE	*Rhinolophus paradoxolophus (R. rex)*	Least Concern	53	0.845	0.619	94.101	6.645	11.067	46.092
RHINOLOPHIDAE	*Rhinolophus pearsonii*	Least Concern	136	0.785	0.474	179.092	10.946	20.959	78.041
RHINOLOPHIDAE	*Rhinolophus pusillus*	Least Concern	173	0.676	0.271	246.343	14.156	26.982	93.739
RHINOLOPHIDAE	*Rhinolophus shameli*	Least Concern	78	0.875	0.658	145.065	7.877	15.734	40.117
RHINOLOPHIDAE	*Rhinolophus siamensis*	Least Concern	25	0.717	0.352	142.418	6.694	14.769	60.924
RHINOLOPHIDAE	*Rhinolophus sinicus*	Least Concern	42	0.787	0.389	142.620	10.939	20.035	71.489
RHINOLOPHIDAE	*Rhinolophus stheno*	Least Concern	59	0.560	0.427	155.281	11.858	22.382	72.898
RHINOLOPHIDAE	*Rhinolophus thomasi*	Least Concern	94	0.799	0.510	147.478	6.384	14.821	62.875

VESPERTILIONIDAE	*Barbastella darjelingensis*	Least Concern	28	0.985	0.936	0.040	0.015	0.025	0.039
VESPERTILIONIDAE	*Eptesicus pachyomus*	Least Concern	37	0.857	0.507	80.585	3.021	5.136	23.470
VESPERTILIONIDAE	*Harpiocephalus harpia*	Least Concern	52	0.786	0.473	113.447	9.687	17.136	61.563
VESPERTILIONIDAE	*Harpiola isodon*	Least Concern	37	0.978	0.874	0.033	0.010	0.013	0.029
VESPERTILIONIDAE	*Hesperoptenus tickelli*	Least Concern	43	0.648	0.237	159.207	6.700	14.577	46.002
VESPERTILIONIDAE	*Hypsugo cadornae*	Least Concern	30	0.742	0.566	107.497	6.934	11.521	49.713
VESPERTILIONIDAE	*Hypsugo pulveratus*	Least Concern	31	0.832	0.636	68.234	4.064	6.533	32.072
VESPERTILIONIDAE	*Ia io*	Near Threatened	39	0.783	0.515	126.820	7.616	12.809	54.113
VESPERTILIONIDAE	*Kerivoula furva*	Least Concern	54	0.982	0.870	2.661	0.025	0.125	1.138
VESPERTILIONIDAE	*Kerivoula hardwickii*	Least Concern	140	0.686	0.285	138.318	12.932	24.523	75.698
VESPERTILIONIDAE	*Kerivoula kachinensis*	Least Concern	24	0.817	0.607	86.608	7.175	15.948	53.230
VESPERTILIONIDAE	*Kerivoula papillosa*	Least Concern	60	0.799	0.450	57.059	6.770	15.194	37.174
VESPERTILIONIDAE	*Kerivoula picta*	Near Threatened	61	0.783	0.571	114.840	2.525	5.493	17.217
VESPERTILIONIDAE	*Kerivoula titania*	Least Concern	35	0.759	0.462	142.020	12.334	23.852	79.938
VESPERTILIONIDAE	*Murina annamitica*	Least Concern	21	0.805	0.625	71.012	8.156	15.190	45.616
VESPERTILIONIDAE	*Murina cyclotis*	Least Concern	130	0.723	0.379	180.535	13.948	26.194	90.020
VESPERTILIONIDAE	*Murina eleryi*	Least Concern	32	0.791	0.654	94.599	7.211	11.371	48.346
VESPERTILIONIDAE	*Murina feae*	Least Concern	55	0.732	0.548	147.935	11.197	20.645	73.531
VESPERTILIONIDAE	*Murina harrisoni*	Least Concern	37	0.746	0.530	125.550	10.425	18.879	67.659
VESPERTILIONIDAE	*Murina huttoni*	Least Concern	48	0.826	0.656	66.870	7.157	14.033	45.212
VESPERTILIONIDAE	*Myotis formosus*	Near Threatened	156	0.900	0.843	26.333	0.245	0.251	0.774
VESPERTILIONIDAE	*Myotis horsfieldii*	Least Concern	107	0.672	0.273	186.076	12.001	23.519	71.639
VESPERTILIONIDAE	*Myotis laniger*	Least Concern	37	0.835	0.526	133.510	8.525	13.811	56.462
VESPERTILIONIDAE	*Myotis muricola*	Least Concern	74	0.619	0.339	168.484	12.382	24.345	73.236

VESPERTILIONIDAE	*Myotis pilosus*	Vulnerable	33	0.865	0.581	66.387	4.879	8.075	35.322
VESPERTILIONIDAE	*Myotis rufoniger*	Least Concern	31	0.915	0.829	6.659	0.365	0.930	2.348
VESPERTILIONIDAE	*Myotis siligorensis (M. alticraniatus)*	Least Concern	80	0.779	0.385	214.202	10.046	20.447	76.933
VESPERTILIONIDAE	*Pipistrellus abramus*	Least Concern	358	0.969	0.840	24.362	0.298	0.300	0.454
VESPERTILIONIDAE	*Pipistrellus ceylonicus*	Least Concern	97	0.897	0.095	80.014	3.056	9.606	24.280
VESPERTILIONIDAE	*Pipistrellus coromandra*	Least Concern	138	0.772	0.472	131.763	8.054	17.524	55.605
VESPERTILIONIDAE	*Pipistrellus javanicus*	Least Concern	96	0.736	0.289	175.610	13.332	25.196	77.854
VESPERTILIONIDAE	*Pipistrellus paterculus*	Least Concern	41	0.830	0.582	62.784	3.844	7.782	32.407
VESPERTILIONIDAE	*Pipistrellus tenuis*	Least Concern	45	0.669	0.303	309.825	16.082	29.111	97.758
VESPERTILIONIDAE	*Scotomanes ornatus*	Least Concern	41	0.882	0.647	81.965	6.412	11.461	42.577
VESPERTILIONIDAE	*Scotophilus heathii*	Least Concern	48	0.722	0.444	151.072	6.506	14.008	57.195
VESPERTILIONIDAE	*Scotophilus kuhlii*	Least Concern	100	0.833	0.635	170.921	3.066	3.399	11.593
VESPERTILIONIDAE	*Tylonycteris robustula*	Least Concern	33	0.754	0.473	103.488	8.483	17.695	47.759
