# Supplementary material for: Habitat prioritization for bat conservation: A case study in Vietnam
Source: PLoS One. 2025 Sep 11;20(9):e0331094. doi: 10.1371/journal.pone.0331094 (PMC12425236; doi:10.1371/journal.pone.0331094)
Supplement: S2 Table — The value measures an environmental variable’s share in the model’s overall gain (a measure of fit). It’s calculated by summing each variable’s incremental gain during the model’s training process and converting these sums to percentages. (PDF) [file pone.0331094.s002.pdf]

Table S2. The contributions of environmental variables in the model of individual species. The values measures an environmental variable's share in the model's overall gain (a measure of fit). It's calculated by summing each variable's incremental gain during the model's training process and converting these sums to percentages.

**Species**	**Bio2***	**Bio10***	**Bio11***	**Bio12***	**Bio18***	**Bio19***	**Crop land**	**Deciduous forest**	**Evergreen forest**	**Flooded vegetation**	**Grassland scrub**	**Urban**	**Karst**
Taphozous melanopogon	0.60	18.87	39.47	0.00	7.89	9.64	10.39	0.00	3.52	5.63	1.92	1.08	1.01
Aselliscus stoliczkanus	2.50	0.00	22.34	1.82	3.28	19.91	0.30	0.30	4.42	0.47	15.83	8.20	20.60
Coelops frithii	5.61	0.00	6.88	0.00	24.83	4.35	12.38	0.49	35.96	5.95	0.00	0.00	3.55
Hipposideros armiger	1.48	4.75	37.72	1.01	22.90	3.04	3.92	2.86	19.43	0.00	1.29	1.50	0.09
Hipposideros cineraceus	11.27	0.38	5.88	0.15	1.72	4.83	12.71	11.58	10.00	0.00	24.20	16.33	0.96
Hipposideros diadema	3.94	0.83	28.17	1.51	7.06	22.04	0.72	0.01	5.92	1.18	0.24	10.07	18.30
Hipposideros galeritus	8.20	1.06	55.76	9.52	3.16	1.86	5.68	0.00	9.21	0.79	0.79	3.90	0.07
Hipposideros gentilis	1.43	2.02	3.33	1.18	7.51	43.57	0.64	0.06	23.84	0.32	12.57	2.00	1.53
Hipposideros larvatus	10.52	0.79	4.95	0.10	24.18	0.83	1.44	0.42	5.87	2.18	18.79	28.34	1.59
Hipposideros lylei	28.98	0.13	5.02	0.24	12.88	15.84	8.42	0.00	6.46	0.35	8.55	2.05	11.08
Lyroderma lyra (L. sinese)	52.26	0.14	9.84	3.13	9.95	7.88	6.34	3.35	4.44	0.00	1.20	1.30	0.17
Megaderma spasma	1.09	0.43	60.26	0.11	3.52	0.41	2.26	0.10	16.44	1.40	2.36	10.51	1.10
Miniopterus magnater	3.91	1.38	0.67	0.35	8.70	6.37	10.57	1.14	25.01	0.00	28.04	5.54	8.31
Miniopterus pusillus	10.94	0.00	1.82	8.69	2.15	0.17	2.56	0.89	11.03	0.07	39.58	22.11	0.00

Chaerephon plicatus (Mops plicatus)	15.03	0.00	18.19	0.00	6.72	0.01	17.37	0.00	20.06	0.38	17.45	4.78	0.00
Cynopterus brachyotis	17.73	5.64	22.22	21.47	3.44	26.38	0.68	0.00	1.08	0.11	0.39	0.86	0.00
Cynopterus sphinx	4.15	9.00	13.58	3.68	8.94	26.47	9.98	0.17	3.37	0.04	1.98	18.58	0.06
Eonycteris spelaea	3.55	2.19	22.40	25.11	5.49	1.07	9.53	0.00	11.54	0.29	5.36	9.56	3.91
Macroglossus minimus	19.43	10.80	13.11	4.75	1.13	28.04	1.24	0.00	14.33	0.23	0.62	6.19	0.15
Macroglossus sobrinus	15.97	0.00	11.47	0.39	23.82	0.05	12.08	0.74	9.46	2.43	19.69	3.92	0.00
Megaerops niphanae	21.21	0.00	6.24	19.28	0.00	14.80	10.82	1.51	1.56	0.07	19.45	5.07	0.00
Pteropus hypomelanus	84.04	0.00	2.67	3.97	2.15	0.00	0.13	0.00	0.00	0.15	1.62	4.00	1.28
Pteropus lylei	4.99	0.08	51.74	1.07	0.00	28.45	5.94	0.57	4.76	0.00	2.17	0.14	0.10
Pteropus vampyrus	0.23	0.10	16.95	3.58	34.12	4.85	3.03	0.01	21.97	1.29	0.00	4.53	9.35
Rousettus amplexicaudatu s	32.97	0.76	23.90	15.40	4.03	1.48	5.28	1.60	2.61	0.54	1.51	6.02	3.91
Rousettus leschenaultii	0.06	0.03	0.61	0.00	0.03	5.50	25.23	4.08	12.41	8.71	31.01	5.36	6.98
Sphaerias blanfordi	6.43	5.74	55.56	0.08	3.97	0.11	3.21	0.50	9.50	0.00	7.19	7.70	0.00
Rhinolophus acuminatus	2.09	0.06	50.57	1.25	3.69	0.34	6.88	0.63	27.00	0.00	5.14	1.45	0.89
Rhinolophus affinis	6.74	1.06	6.83	0.19	25.51	0.89	1.47	1.61	29.59	1.83	11.23	13.01	0.03
Rhinolophus borneensis (R. chaseni)	2.78	0.84	3.96	0.09	6.51	60.42	2.44	0.00	11.32	0.00	0.03	2.92	8.70
Rhinolophus lepidus	36.60	0.29	0.00	10.86	17.79	1.76	8.58	1.92	5.44	0.00	13.83	0.00	2.94

Rhinolophus luctus	26.45	0.90	0.62	2.07	1.51	2.15	1.90	1.42	40.97	1.21	11.06	0.14	9.61
Rhinolophus macrotis (R. episcopus)	1.77	4.19	4.44	0.93	0.15	3.36	6.96	1.76	18.15	2.62	13.35	12.58	29.74
Rhinolophus malayanus	4.86	1.00	9.50	0.18	11.51	27.29	1.95	3.48	8.67	1.84	7.42	21.12	1.19
Rhinolophus marshalli	3.69	0.01	6.03	2.08	6.10	31.15	5.46	5.67	3.18	0.42	19.96	12.62	3.66
Rhinolophus microglobosus	15.91	2.16	6.78	0.01	5.35	32.67	1.24	1.98	8.67	0.12	12.58	12.55	0.00
Rhinolophus paradoxolophus (R. rex)	2.17	0.45	25.97	4.46	8.96	11.93	0.48	0.00	3.66	0.00	12.73	1.97	27.21
Rhinolophus pearsonii	15.38	0.22	32.31	0.97	11.69	13.36	4.98	0.16	15.91	0.00	0.37	0.15	4.52
Rhinolophus pusillus	13.50	0.22	0.00	1.27	5.44	34.28	2.43	0.57	20.10	0.47	19.90	1.82	0.00
Rhinolophus shameli	0.79	0.00	25.42	7.23	12.21	31.99	4.11	0.56	10.13	0.29	1.01	6.25	0.00
Rhinolophus siamensis	0.38	0.24	10.28	0.01	3.30	18.01	0.00	5.20	6.38	0.00	33.30	13.42	9.48
Rhinolophus sinicus	0.12	0.05	30.85	2.54	24.19	12.56	1.32	0.00	24.28	0.73	3.33	0.00	0.02
Rhinolophus stheno	13.97	0.06	6.90	21.65	4.54	0.32	2.58	5.03	28.77	0.06	10.40	5.61	0.09
Rhinolophus thomasi	16.66	1.23	0.30	3.22	3.66	26.50	1.35	0.12	7.16	0.08	11.89	10.18	17.64
Barbastella darjelingensis	0.00	9.40	47.51	0.00	13.22	19.71	0.00	0.33	9.26	0.00	0.57	0.00	0.00
Eptesicus pachyomus	0.00	1.89	43.21	0.00	3.54	24.00	20.45	0.00	3.98	1.02	1.92	0.00	0.00
Harpiocephalus harpia	1.20	1.47	24.74	0.41	16.01	0.32	1.29	1.03	36.36	0.00	1.05	6.64	9.48
Harpiola isodon	4.16	1.73	41.96	0.00	18.72	19.21	1.58	0.23	12.01	0.00	0.10	0.28	0.03

Hesperoptenus tickelli	13.71	0.00	13.51	0.76	16.36	0.00	8.04	3.76	4.89	1.10	28.22	6.22	3.41
Hypsugo cadornae	0.00	0.13	37.74	1.98	1.05	11.39	10.30	1.16	6.81	0.35	9.52	2.50	17.06
Hypsugo pulveratus	0.04	0.02	7.65	3.50	4.72	24.77	0.43	0.74	5.60	0.04	1.37	3.92	47.19
Ia io	3.08	0.00	37.24	0.00	3.27	6.05	3.90	4.84	7.25	0.04	9.78	0.00	24.55
Kerivoula furva	0.99	2.50	27.76	9.53	26.52	22.21	0.43	0.30	8.36	0.25	0.00	0.55	0.60
Kerivoula hardwickii	2.07	0.67	9.86	7.59	7.86	3.91	18.89	0.24	24.34	0.31	5.11	15.56	3.57
Kerivoula kachinensis	5.67	1.13	8.20	0.00	3.33	34.83	17.64	6.11	3.36	4.90	2.95	11.76	0.12
Kerivoula papillosa	4.51	1.62	21.26	0.00	1.83	10.28	3.09	1.23	33.06	2.39	1.27	7.29	12.18
Kerivoula picta	6.32	0.00	5.44	1.24	16.61	0.87	14.86	1.82	40.18	0.00	4.02	4.14	4.51
Kerivoula titania	17.85	0.45	13.27	3.06	2.36	0.00	28.37	6.74	12.36	0.00	0.00	14.96	0.57
Murina annamitica	10.34	0.00	8.33	0.00	13.76	4.44	27.32	2.86	28.41	0.00	0.00	4.52	0.02
Murina cyclotis	7.98	0.35	4.40	10.26	4.67	0.82	10.55	1.34	32.95	0.00	9.69	14.92	2.07
Murina eleryi	20.51	1.12	47.71	0.00	1.33	0.00	6.23	2.65	7.91	4.16	0.25	8.13	0.00
Murina feae	17.17	0.40	9.20	1.48	3.19	19.98	4.01	1.85	32.38	0.00	3.46	6.85	0.03
Murina harrisoni	3.01	0.00	0.12	7.74	0.00	37.86	12.52	1.79	16.88	0.39	6.79	12.36	0.56
Murina huttoni	7.45	15.94	45.73	5.86	0.80	1.74	7.30	0.00	13.89	0.00	0.89	0.07	0.34
Myotis formosus	0.38	0.01	25.88	3.79	14.03	5.70	34.27	0.00	8.17	0.06	7.24	0.44	0.02
Myotis horsfieldii	3.02	0.26	11.79	21.15	9.06	1.08	6.14	8.38	10.99	0.00	8.73	15.21	4.18
Myotis laniger	1.04	0.65	61.46	0.06	6.07	0.00	1.47	2.83	10.91	0.96	0.17	8.27	6.11
Myotis muricola	6.20	0.53	10.71	2.29	12.64	0.22	8.75	7.64	29.00	1.95	5.29	14.76	0.00
Myotis pilosus	0.00	0.00	26.34	1.83	0.99	16.99	5.77	0.00	2.95	0.00	2.74	2.05	40.35
Myotis rufoniger	5.06	0.87	66.12	7.77	0.27	5.84	3.37	0.32	8.16	0.18	0.14	0.57	1.33
Myotis siligorensis	5.71	0.33	0.25	1.21	6.75	22.99	4.33	0.93	2.25	0.02	18.50	14.60	22.13

(M. alticraniatus)													
Pipistrellus abramus	0.81	1.47	60.42	1.76	5.35	3.07	2.98	0.51	7.74	0.68	10.93	1.12	3.17
Pipistrellus ceylonicus	8.63	0.57	8.10	0.84	56.73	15.39	1.96	0.25	2.89	0.33	1.00	1.14	2.18
Pipistrellus coromandra	64.54	4.87	10.74	0.19	4.07	2.02	6.40	0.00	2.91	0.06	0.96	0.39	2.84
Pipistrellus javanicus	7.25	17.33	5.68	3.16	6.59	4.95	5.41	0.09	43.33	1.25	2.73	2.20	0.03
Pipistrellus paterculus	13.84	0.98	0.00	0.85	10.49	58.44	3.60	1.95	0.49	0.00	6.80	2.04	0.52
Pipistrellus tenuis	0.97	0.00	8.50	14.49	2.71	17.49	21.42	2.53	7.16	0.00	7.26	14.63	2.85
Scotomanes ornatus	4.66	0.41	37.76	0.00	2.53	14.34	0.36	2.29	32.65	0.00	1.23	3.69	0.08
Scotophilus heathii	28.78	0.17	1.98	0.41	0.01	45.10	5.25	1.55	0.00	0.01	14.07	2.10	0.56
Scotophilus kuhlii	18.91	4.43	3.29	1.38	1.52	1.02	35.76	0.00	9.42	0.13	11.78	2.73	9.64
Tylonycteris robustula	9.07	0.00	27.97	6.64	11.82	1.35	6.81	3.03	14.21	0.00	4.06	15.05	0.00

* Bio2: Mean diurnal range; Bio10: Mean temperature of warmest quarter; Bio11: Mean temperature of coldest quarter; Bio12: Annual precipitation; Bio18: Precipitation of warmest quarter; Bio19: Precipitation of coldest quarter
