# Supplementary material for: Habitat prioritization for bat conservation: A case study in Vietnam
Source: PLoS One. 2025 Sep 11;20(9):e0331094. doi: 10.1371/journal.pone.0331094 (PMC12425236; doi:10.1371/journal.pone.0331094)
Supplement: S3 Table — The values were calculated from the data in the World Database on Protected Areas. The corresponding national categories are also provided. (PDF) [file pone.0331094.s003.pdf]

Table S3. The number and total area of protected areas in Vietnam by the IUCN category. The values were calculated from the data in the World Database on Protected Areas (The Global Database on Protected Areas Management Effectiveness (GD-PAME), 11/2023, Cambridge, UK: UNEP-WCMC and IUCN. Available at: www.protectedplanet.net). The corresponding national categories are also provided.

IUCN category	Vietnam category	Number of areas	Area (km^2)
II	National Parks	27	12,813
IV	Nature Reserves	37	10,623
V	Cultural and Historical Sites	25	1,795
Total		89	25,231
