# Supplementary material for: Habitat prioritization for bat conservation: A case study in Vietnam
Source: PLoS One. 2025 Sep 11;20(9):e0331094. doi: 10.1371/journal.pone.0331094 (PMC12425236; doi:10.1371/journal.pone.0331094)
Supplement: S1 Fig — The values shown are Spearman’s rank correlation coefficients. (PDF) [file pone.0331094.s004.pdf]

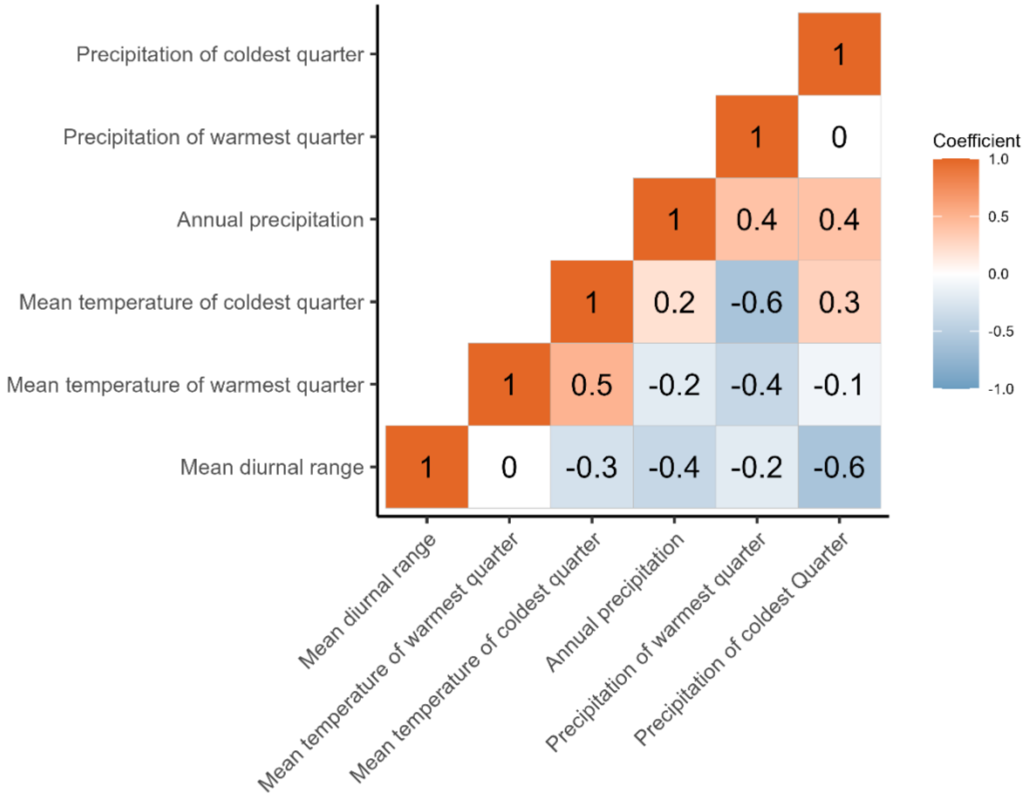

Figure S1. Pairwise correlations of the six bioclimatic variables used for modeling the distributions of Vietnamese bat species. The values shown are Spearman's rank correlation coefficients.
