# Supplementary material for: Habitat prioritization for bat conservation: A case study in Vietnam
Source: PLoS One. 2025 Sep 11;20(9):e0331094. doi: 10.1371/journal.pone.0331094 (PMC12425236; doi:10.1371/journal.pone.0331094)
Supplement: S2 Fig — Each boxplot shows the minimum, 25th percentile, median, 75th percentile and maximum values across all species modeled in the study. The boxplots and associated combinations are ordered by the AUC values, with those on the top having the highest value. The feature types are linear (L), quadratic (Q) and hinge (H). (PDF) [file pone.0331094.s005.pdf]

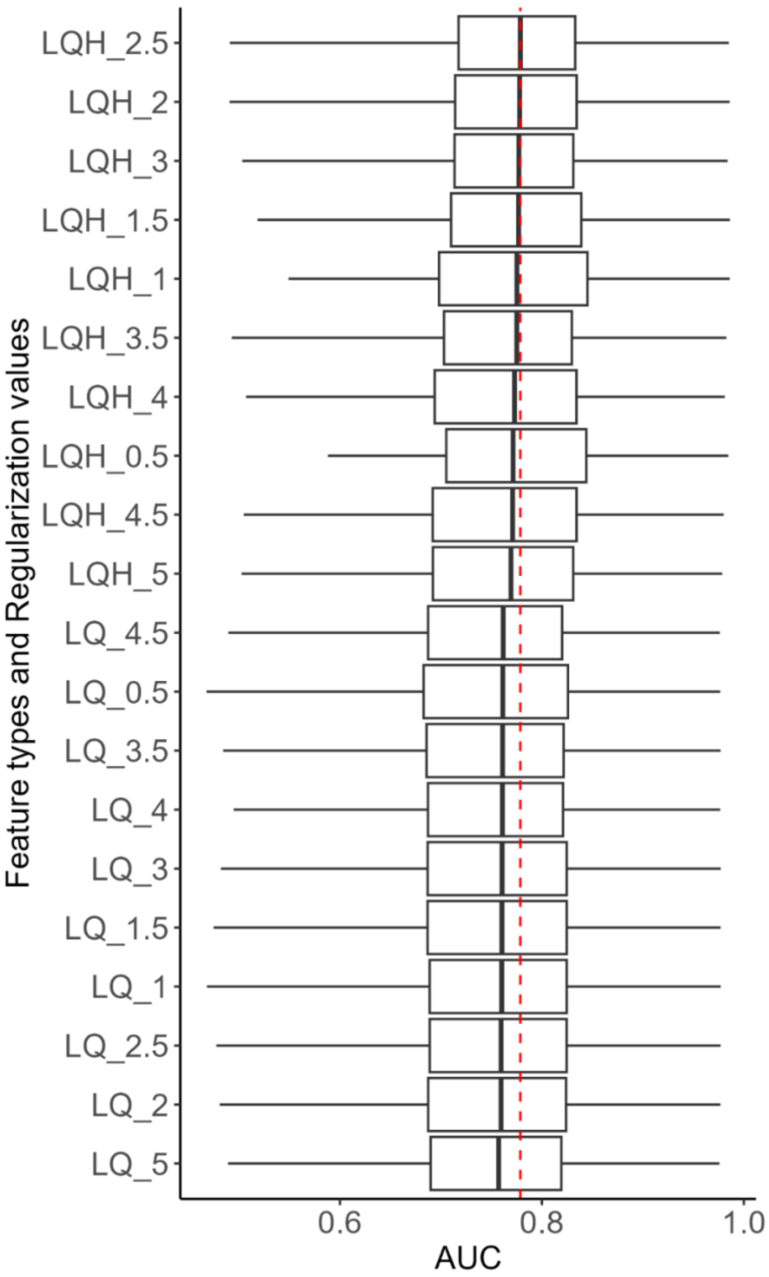

Figure S2. The AUC values of the distribution models built for individual bat species with different combinations of feature types and regularization values. Each boxplot shows the minimum, 25th percentile, median, 75th percentile and maximum values across all species modeled in the study. The boxplots and associated combinations are ordered by the AUC values, with those on the top having the highest value. The feature types are linear (L), quadratic (Q) and hinge (H).
