# Supplementary material for: Habitat prioritization for bat conservation: A case study in Vietnam
Source: PLoS One. 2025 Sep 11;20(9):e0331094. doi: 10.1371/journal.pone.0331094 (PMC12425236; doi:10.1371/journal.pone.0331094)
Supplement: S3 Fig — The red line shows the value of 0.7. (PDF) [file pone.0331094.s006.pdf]

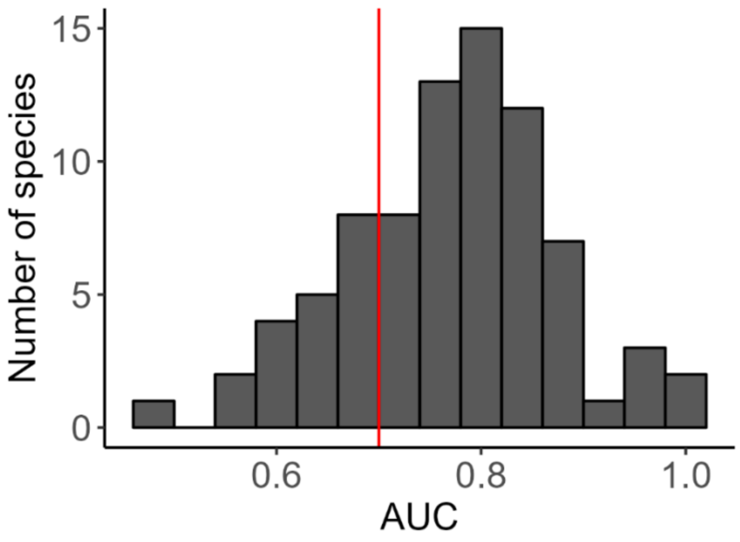

Figure S3. AUC values of the distribution models built for the bat species in Vietnam. The red line shows the value of 0.7.
