# Supplementary material for: Habitat prioritization for bat conservation: A case study in Vietnam
Source: PLoS One. 2025 Sep 11;20(9):e0331094. doi: 10.1371/journal.pone.0331094 (PMC12425236; doi:10.1371/journal.pone.0331094)
Supplement: S4 Fig — Each boxplot shows the minimum, 25th percentile, median, 75th percentile and maximum values across all species modelled in the study. The variables are ordered by the medians of the percent contributions, with the one on the top having the highest median. (PDF) [file pone.0331094.s007.pdf]

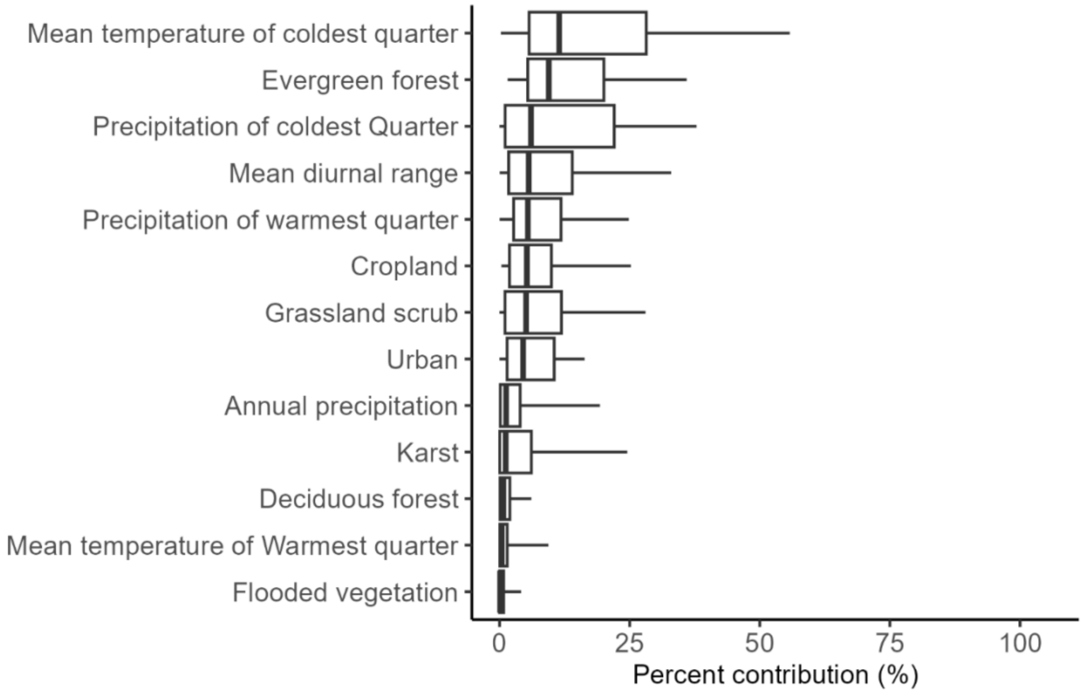

Figure S4. The percent contributions of the environmental variables across the bat species distribution models. Each boxplot shows the minimum, 25th percentile, median, 75th percentile and maximum values across all species modelled in the study. The variables are ordered by the medians of the percent contributions, with the one on the top having the highest median.
