# Supplementary material for: Habitat prioritization for bat conservation: A case study in Vietnam
Source: PLoS One. 2025 Sep 11;20(9):e0331094. doi: 10.1371/journal.pone.0331094 (PMC12425236; doi:10.1371/journal.pone.0331094)
Supplement: S5 Fig — Same as Fig 3, but for bat species with an AUC value greater than 0.7. (PDF) [file pone.0331094.s008.pdf]

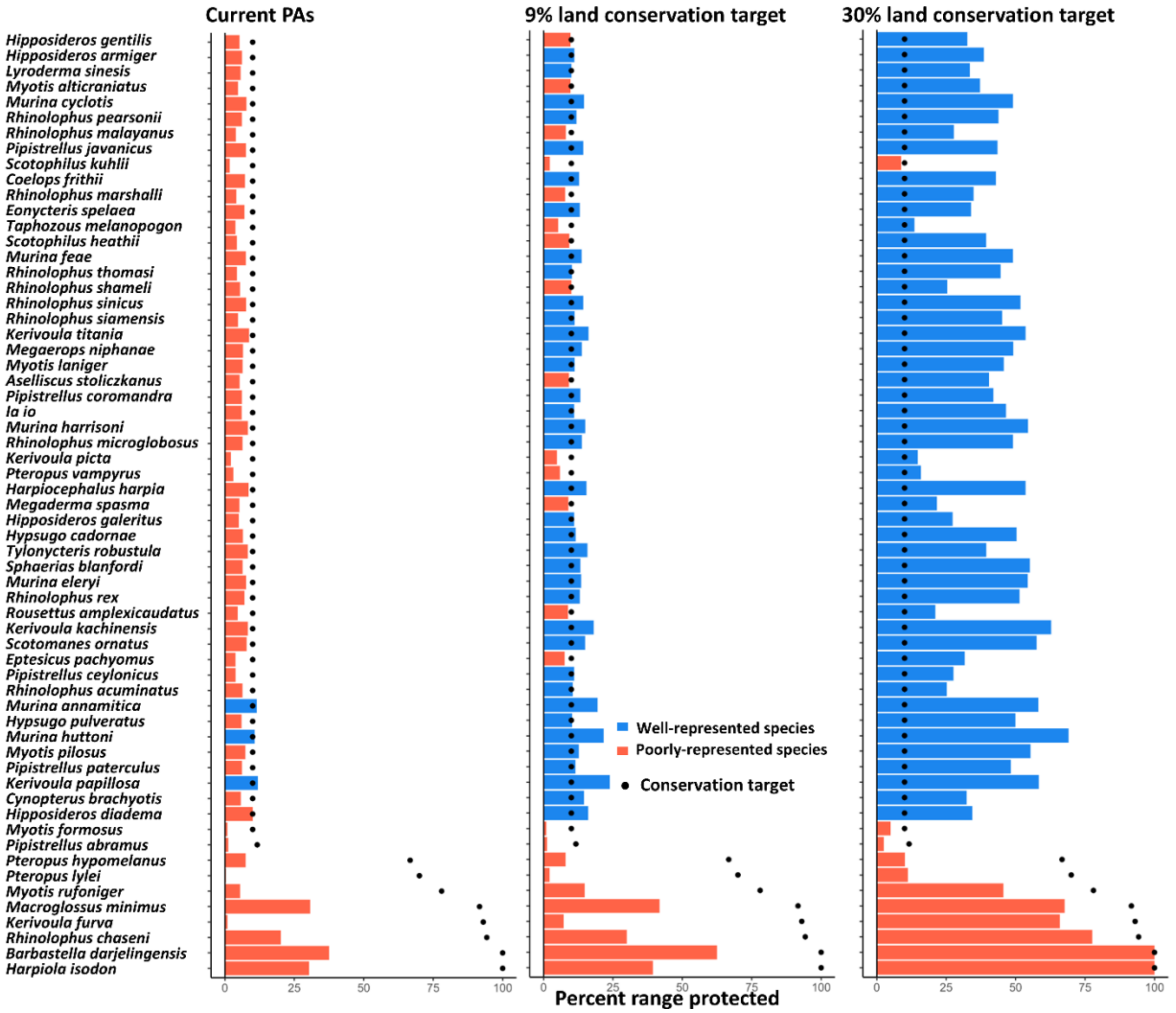

Figure S5. Representativeness of current protected areas, and the prioritized areas with the conservation targets of 9% and 30% land coverage. Same as Figure 3, but for bat species with an AUC value greater than 0.7.
