# Supplementary material for: Habitat prioritization for bat conservation: A case study in Vietnam
Source: PLoS One. 2025 Sep 11;20(9):e0331094. doi: 10.1371/journal.pone.0331094 (PMC12425236; doi:10.1371/journal.pone.0331094)
Supplement: S6 Fig — Same as Fig 4, but for the bat species with an AUC value greater than 0.7. The protected area boundaries were obtained from the World Database on Protected Areas [34]. Copyright notice: © UNEP-WCWC and IUCN – WDPA 2023. (PDF) [file pone.0331094.s009.pdf]

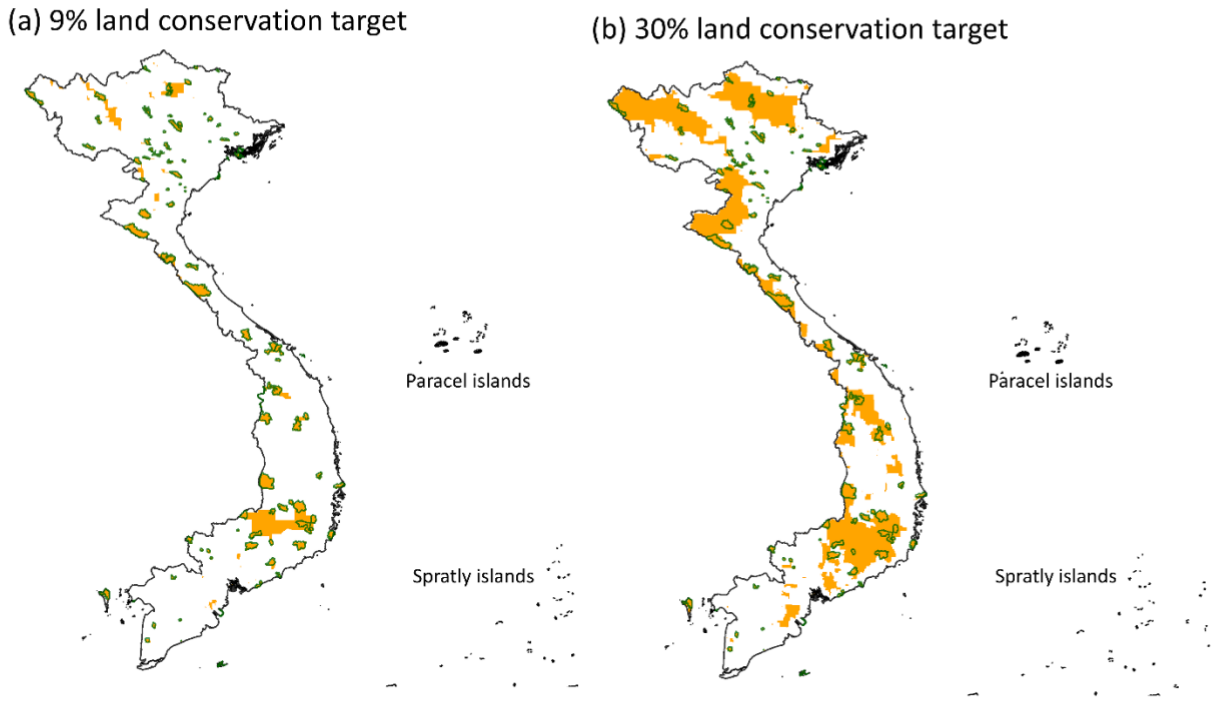

Figure S6. Distribution of prioritized conservation areas for bat species in Vietnam identified by the spatial prioritization analysis. Same as Figure 4, but for the bat species with an AUC value greater than 0.7. The protected area boundaries were obtained from the World Database on Protected Areas [34]. **Copyright notice**: © UNEP-WCWC and IUCN – WDPA 2023
